# Customized Utilization Strategies of Industrial Lignin to Produce Adsorbents and Flocculants Based on Fractionation and Adequate Structural Interpretation

**DOI:** 10.3390/ijms23126617

**Published:** 2022-06-14

**Authors:** Lei Wang, Dewei Yang, Xiaohan Li, Xinyi Zhu, Jungang Jiang, Yifan Zhang, Xue Chen, Hongbo Yu

**Affiliations:** 1 Hubei Provincial Key Laboratory of Green Materials for Light Industry, School of Materials and Chemical Engineering, Hubei University of Technology, Wuhan 430068, China; y13235634365@163.com (D.Y.); lxiaohan0317@163.com (X.L.); 102000414@hbut.edu.cn (X.Z.); jungang.jiang@hbut.edu.cn (J.J.); zhangyifan@hbut.edu.cn (Y.Z.); 2 School of Engineering, Jining University, Qufu 273155, China; chenxue37121@163.com; 3 Key Laboratory of Molecular Biophysics of MOE, College of Life Science and Technology, Huazhong University of Science and Technology, Wuhan 430074, China; yuhongbo@hust.edu.cn

**Keywords:** fractionation, lignin-based flocculants, lignin-based activated carbons, purification mechanism, wastewater resources

## Abstract

Lignin, a by-product of pulping and biorefinery, has great potential to replace petrochemical resources for wastewater purification. However, the defects of lignin, such as severe heterogeneity, inferior reactivity and poor solubility, characterize the production process of lignin-based products by high energy consumption and serious pollution. In this study, several lignin fractions with relatively homogeneous structure were first obtained by organic solvent fractionation, and their structures were fully deciphered by various characterization techniques. Subsequently, each lignin component was custom-valued for wastewater purification based on their structural characteristics. Benefiting from the high reactivity and reaction accessibility, the lignin fraction (lignin-1) refined by dissolving in ethanol and n-butanol could been used as a raw material to produce cationic lignin-based flocculant (LBF) in a copolymerization system using green, cheap and recyclable ethanol as solvent. The lignin fraction (lignin-2) extracted by methanol and dioxane showed low reactivity and high carbon content, which was used to produce lignin-based activated carbon (LAC) with phosphoric acid as activator. Moreover, the influences of synthetic factors on the purification capacity were discussed, and the LBF and LAC produced under the optimal conditions showed distinguished purification effect on kaolin suspension and heavy metal wastewater, respectively. Furthermore, the corresponding purification mechanism and external factors were also elaborated. It is believed that this cleaner production strategy is helpful for the valorization of lignin in wastewater resources.

## 1. Introduction

The improvement of living quality and the rapid development of industrial technologies are making the composition of actual wastewater increasingly complex, coupled with the rapid growth of the population, resulting in a serious water crisis [1]. Wastewater recycling is an effective way to solve the water crisis [2]. Flocculation and adsorption are indispensable steps to purify wastewater, and the development of efficient, green and sustainable flocculants and adsorbents is crucial [3]. Considering the increasingly serious environmental problems and the depletion of petrochemical resources, it is urgent to develop natural polymer-based materials featuring high efficiency, low cost, raw material regeneration, and no secondary pollution for wastewater recycling.

Lignin, the most abundant renewable aromatic resource, is a high-volume by-product arising from pulping and biorefinery [4]. Lignin has a high amount of reactive functional groups (e.g., -OH, -COOH, and -OCH_3_), a rigid core skeleton and high carbon content, which give it great potential to produce flocculants and adsorbents [5,6]. Research on the application of lignin in flocculants [7], adsorbents [8], hydrogels [9], composites [10], and thermoplastics [11] has been carried out for a long time. However, great breakthroughs have not been made in the industrial process of lignin-based materials in spite of large quantities of studies, which is mainly due to the severe heterogeneity of lignin caused by plant species, biosynthesis and extraction processes [12,13].

Reducing the heterogeneity of lignin and customizing the utilization according to the structural characteristics of each component are effective strategies to overcome the aforementioned problems. To make lignin more uniform, several fractionation methods have been developed [14]. However, the pH acidic precipitation method, membrane separation method and gel permeation chromatography method have the disadvantages of low efficiency [15], high cost due to a high amount of filter membranes [16], and complex equipment [17], respectively. The organic solvent fractionation has the advantages of easy operation, low costs, and recoverable solvents [18]. Several lignin fractions with homogeneous structures can be obtained by organic solvent fractionation in accordance with physicochemical properties [19,20]. Moreover, after fully deciphering the structure of each lignin component, they can be custom applied according to their structural characteristics, which greatly promotes the development of lignin-based products. Lignin refined by sequential solvent fractionation has been successfully used for the production of lignin-based epoxy resins owing to its relatively low dispersion [12]. Unfortunately, only part of the fractionated lignin fraction was used in these studies, and there are no reports on the utilization of the remaining lignin fraction. Furthermore, there is little research on the utilization of fractionated lignin in wastewater resources.

In order to solve the problems of poor reactivity and solubility of lignin, dioxane, dimethyl sulfoxide and high-concentration alkali liquor are usually used as reaction solvents in the production of lignin-based flocculants [21,22]. These reaction solvents can cause serious environmental burden and high cost [23]. Ethanol as a reaction solvent has excellent application prospects due to its low price, non-toxicity, and easy recovery [24]. However, most industrial lignin is only partially soluble in ethanol, limiting the application of ethanol as a solvent to produce lignin-based flocculants [25]. Therefore, refining to obtain lignin components that can be completely dissolved in ethanol and have high reactivity is a practical idea for realizing the green synthesis of lignin-based flocculants in ethanol solution. Note that the lignin components remaining after refining with the above requirements usually have large molecular weight and high carbon content, which are suitable as precursors for the production of lignin-based activated carbons.

In this study, industrial alkali lignin was first divided into several more homogeneous lignin components by organic solvent fractionation, and their structures were adequately deciphered. Subsequently, according to the structural characteristics, fractionated lignin components have been used as raw materials to produce different materials for purifying wastewater. The lignin-based flocculant was produced in the copolymerization system where a lignin component with superior solubility and reactivity was used as raw material, trimethyl-2-methacroyloxyethylammonium chloride as the monomer, and ethanol as the solvent, and the kaolin suspension was employed to test its flocculation performance. Lignin-based activated carbon was produced using lignin components with larger molecular weight and higher carbon content as precursors for the adsorption of heavy metals in wastewater. Moreover, the purification mechanisms of lignin-based flocculant and lignin-based activated carbon in the purification of wastewater were systematically explored. Furthermore, the influences of external factors on the purification effects were discussed in detail.

## 2. Results and Discussion

### 2.1. Classification of Lignin and the Characterization of its Components

Considering the effects of binding energy and hydrogen bonding capacity for lignin-dissolving properties in organic solvents, four organic solvents including n-butanol, ethanol, methanol, and dioxane were selected. As shown in Table 1, the yields of F1-F5 were 15%, 34.93%, 19.10%, 21.22%, and 9.15%, respectively. Before fractionation, KL showed a molecular weight (2777 g/mol) with a PDI up to 3.15. The fractionated lignin components (F1-F4) were more homogeneous, and their Mw increased sequentially, at 1141, 2696, 3987, 6794, and 9820 g/mol, respectively. Notably, the phenolic hydroxyl content of lignin decreased as its molecular weight increased, which was because the cleavage of the ether bond produced more phenolic hydroxyl groups. F1 and F2 had higher phenolic hydroxyl content than KL, which were more suitable to produce lignin-based products by free radical polymerization.

Structural information for all samples obtained by FT-IR is shown in Figure S1. The main signals appear at 1598, 1508, and 1420 cm^−1^ (aromatic skeletal vibrations), which indicated that the lignin retains its typical structure [26]. However, lignin with higher molecular weight had lower relative strength of unconjugated carbonyl stretching at 1712 cm^−1^ [26]. This phenomenon revealed that the carboxyl groups in lignin with a decrease were favored by a higher molecular weight, which was consistent with that of ^31^P NMR.

**Figure 1:**
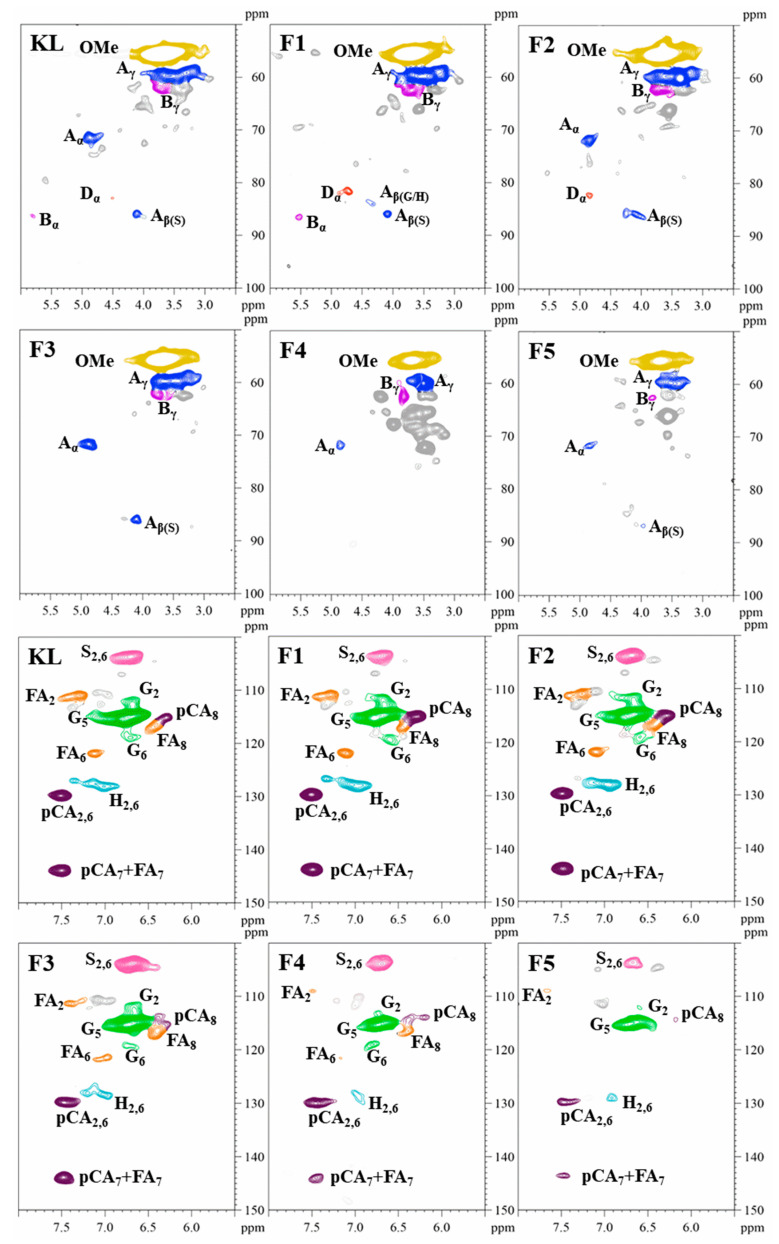
The side chain region (δ_C_/δ_H_ 50–100/2.5–6.0) and aromatic region (δ_C_/δ_H_ 100–150/5.5–8.0) in the 2D HSQC NMR spectra of lignin samples. The alkali lignin (KL) isolated from corn cob, the lignin fractions collected from n-butanol, ethanol, methanol, and dioxane were referred as F1, F2, F3, and F4, respectively, and F5 was an insoluble residue.

Figure 1 and Figure S2 show the 2D-HSQC NMR spectra of all lignin samples, and the corresponding cross-signals refer to previous studies [27,28,29]. In terms of the side chain region, it could be observed that β-*O*-4 was the main linkage between the lignin units. The lignin component with a stronger β-*O*-4 signal displayed a larger molecular weight, which was consistent with the results of previous studies [30]. Moreover, the cleavage of the β-*O*-4 linkage might generate phenolic hydroxyl groups, therefore the lignin component with a small molecular weight showed a higher content of phenolic hydroxyl groups [31]. In addition, no cross-linking signals related to carbohydrates were observed in all lignin samples, indicating that these lignin samples were pure and had good application prospects. For the aromatic region, in addition to the cross-linking signals attributed to S, G, and H units, a distinct signal for dissociative *p*-coumaric acid (*p*-CA) was also observed, showing a typical lignin structure isolated from corn cob. It was found that the proportion of lignin-containing S units with an increase is favored by a higher molecular weight. Considering the active site and steric hindrance, the lignin consisting of low molecular weight had relatively more H and G units and exhibited higher reactivity.

Combined with the above analysis, F1 and F2 with lower molecular weight showed more Ph-OH groups. Therefore, lignin-1 (F1 and F2) used as a raw material showed noticeable benefits for the production of lignin-based flocculant. The lignin-2 (F3 and F4) had higher molecular weight and fewer phenolic hydroxyl (Ph-OH) and carboxyl groups (-COOH), resulting from higher carbon content. They were suitable as precursors for the production of lignin-based activated carbon. F5 accounted for a small proportion and contained a large number of impurities, so it was not included in the follow-up study.

### 2.2. Production of Lignin-Based Flocculant

The polymerization mechanism is shown in Scheme S1. First, AIBN was decomposed to produce primary free radicals at high temperature. The phenolic hydroxyl group of lignin was attacked by primary free radicals, producing phenolic oxygen radicals. Meanwhile, these primary radicals attacked the alkenyl groups of METAC and produced METAC radicals, finally obtaining a lignin-based flocculant [32,33]. In addition, the PMETAC was produced as a side effect by polymerization between METAC radicals (as shown in Scheme S1B).

The flocculation property of lignin-based flocculant is determined by a variety of internal factors, for example hydrophilicity, charge density, and molecular weight, which are determined by the production conditions [34]. Therefore, production conditions such as the dosage ratio of MEATC and lignin, reaction time and reaction temperature were performed in this study. The synthesis process and structural information of lignin-based flocculants were recorded in Table S1.

**Figure 2:**
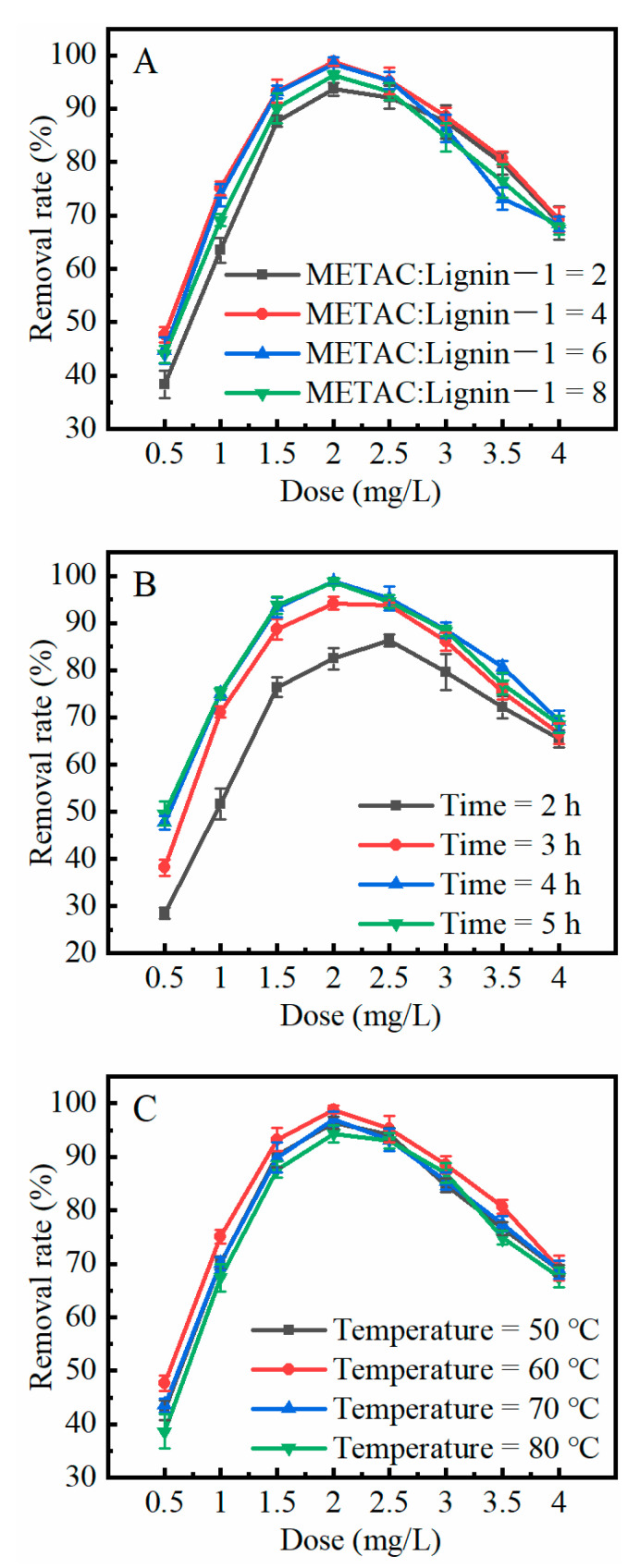
Effect of production conditions on removal rate of lignin-based flocculant: (**A**) impregnation ratio, (**B**) time, and (**C**) temperature. Error bars represent the standard deviation from three repetitions.

The ratio of MEATC to lignin shows an effect on the flocculation performance of lignin-based flocculant (Figure 2A). As the METAC dosage increased, the removal of kaolin suspension by lignin-based flocculant first increased and then decreased. This was because more METAC monomer could increase the grafting rate of lignin, so that more METAC was grafted onto the lignin backbone. Therefore, the lignin-based flocculant produced under the larger METAC dosage ratio had higher molecular weight and positive charge density, exhibiting better flocculation performance. However, an excess of METAC monomers would cause a large number of homo-polymerization reactions, thereby reducing the grafting rate and flocculation property of lignin-based flocculant [33]. The effect of reaction time on flocculation ability of lignin-based flocculant is shown in Figure 2B. The flocculation property of lignin-based flocculant improved rapidly as reaction time increased (from 2 h to 4 h). A longer time was helpful for the sufficient progress of the copolymerization of lignin and METAC. However, the flocculation property of lignin-based flocculant hardly changed when the reaction time exceeded 4 h. Furthermore, the flocculation property of lignin-based flocculant affected through change in reaction time was also investigated (Figure 2C). The degree of substitution of lignin increased as temperature increased at the initial stage, and the flocculation ability of lignin-based flocculant was improved. This phenomenon suggested that the polymerization reaction was an endothermic reaction, namely that the higher temperature facilitated the excitation of free radicals and the polymerization between free radicals, yet at the same time the chain transfer reaction and chain termination reaction hindered the copolymerization of lignin and METAC at high temperature over 60 °C [35]. Regarding the above discussion, the optimum production conditions of lignin-based flocculant (LBF) for flocculating kaolin were as follows: ratio of MEATC and lignin of 4, reaction time of 4 h and reaction temperature of 60 °C. The production of LBF was performed using the above conditions as the object of subsequent research.

### 2.3. Characterization of Lignin-Based Flocculant

**Figure 3:**
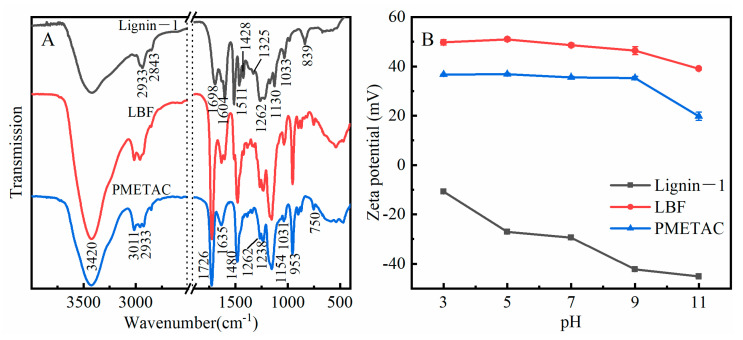
(**A**) FTIR spectra of Lignin-1, PMETAC and LBF, (**B**) zeta potential of Lignin-1, PMETAC and LBF as function of the pH.

The FT-IR spectra of lignin-1, LBF, and PMETAC are shown in Figure 3A. The signals detected at 1604, 1511 and 1428 cm^−1^ on lignin-1 and LBF are attributed to the characteristic peak of lignin aromatic skeleton, and new peaks for LBF that occurred at 1726, 1480, and 953 cm^−1^ are primary derived from the C=O, C-N, and -OCH_3_ of quaternary ammonium, respectively [26,36,37]. These characteristic signals were agreed with the assignment of PMETAC FT-IR bands, which preliminarily proved that PMETAC can be grafted into the skeleton of lignin. Moreover, the signal intensity attributed to the phenolic hydroxyl group in lignin was weakened at 1033 cm^−1^, confirming that the copolymerization took place on the phenolic hydroxyl group of lignin [37].

Figure 3B compares the zeta potentials of lignin-1, LBF, and PMETAC at different pH values [38]. The measurements showed that the zeta potentials of lignin-1 in solutions with pH values between 3 and 11 were negative because of the negatively charged groups (carboxyl group and phenolic hydroxyl groups). However, after copolymerization, the cationic group of METAC was grafted to lignin. Therefore, the LBF exhibited a higher positive charge density, and showed positive zeta potentials over all pH ranges. It was worth noting that the lignin skeleton provided a rigid core for LBF, which enabled LBF to have a more stable form in water. Therefore, the LBF has a higher positive charge density than PMETAC, which proved further that METAC had been successfully grafted into the skeleton of lignin.

### 2.4. External Factors Influencing the Flocculation Effect

**Figure 4:**
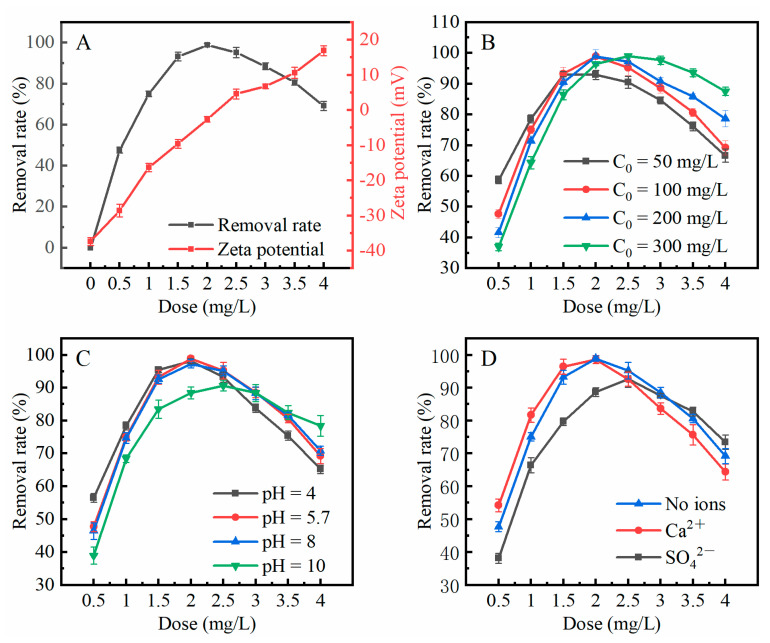
Effects of (**A**) dose, (**B**) initial concentration of the kaolin suspension, (**C**) suspension pH and (**D**) coexisting ions on the removal rate. Error bars represent the standard deviation from three repetitions.

Investigating the effect of external factors on the flocculation efficiency can evaluate the commercial prospects of LBF. Figure 4A shows the effect of the LBF dosage on the removal rate of the kaolin suspension and the zeta potential of the corresponding supernatant. The removal rate of kaolin suspension went up first with increases in LBF dosage and then decreased, and the best turbidity removal rate of LBF for kaolin suspension was 98.87% at 2 mg/L dosage. In the initial increasing stage of LBF dosage, since the kaolin suspension contains a number of negative charges, the addition of more LBF with positive charges could destabilize the kaolin particles under the action of electrostatic attraction, thereby promoting the flocculation effect. At this time, a number of negative charges in the kaolin suspension were rapidly neutralized, so the zeta potential increased rapidly. Until optimal flocculation was achieved, the potential of the supernatant approached zero, which was consistent with the classical charge neutralization theory [39]. As the dosage of LBF continued to increase, the increase of the zeta potential resulted in lower removal rate. This was because LBF increased the electrostatic repulsion between particles, resulting in a re-stabilization phenomenon.

Figure 4B shows the effect of the initial kaolin concentration (C_0_) on the removal rate, and LBF showed significant flocculation effects at all four different initial concentrations of kaolin suspension. As the C_0_ increased, the total amount of negative charge also increased, therefore a larger dosage of LBF was required. However, the optimal dosage was far lower than the theoretical calculated value, which was a typical flocculation system dominated by bridging [40,41,42].

The effect of kaolin suspension pH on the removal rate is shown in Figure 4C. When the suspension pH was in the range of 4 to 8, the removal rate of LBF to the kaolin suspension was higher than 95% at the dosage of 2 mg/L, which showed a satisfactory adaptability to the wastewater pH. Under acidic conditions, the flocculation effect of LBF was slightly enhanced due to protonation. When wastewater pH was 10, LBF eliminated 90% of kaolin suspension at the optimal dosages of 2.5 mg/L. This was because many hydroxide ions reduced the electrostatic attraction of LBF through deprotonation, thereby reducing the flocculation ability of LBF [43].

The influences of coexisting ions Ca^2+^ and SO_4_^2−^ on the flocculation efficiencies of LBA were studied (Figure 4D). The results showed that the flocculation effect of LBF was slightly improved by adding a high concentration of Ca^2+^. The presence of Ca^2+^ could strengthen the electrostatic attraction between LBF and kaolin particles, thereby promoting the formation of flocs and increasing the flocculation effect. Conversely, the presence of SO_4_^2−^ weakened the electrostatic attraction, reduced the flocculation effect and increased the consumption of LBF. However, LBF exhibited outstanding flocculation effect whether it was faced with wastewater with high concentration of anions or cations. In general, LBF could be applied to various complex external factors, such as wastewater with different initial concentrations, a wide range of pH and coexisting ions.

**Scheme 1:**
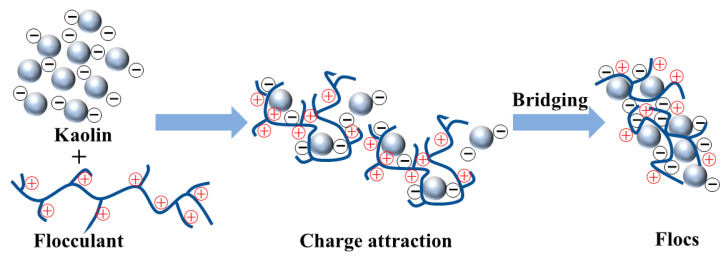
Flocculation mechanism of lignin-based flocculant on kaolin suspension.

Scheme 1 shows the flocculation mechanism of LBF on the kaolin suspension. The kaolin particle surface was negatively charged, and the LBF had positive charge, so the flocculation process in the kaolin suspension treatment was mainly charge attraction, and then the flocculation group was formed through bridging [32,42].

### 2.5. Production and Characterization of the Lignin-Based Activated Carbon

Table S2 shows the BET of lignin-based activated carbon produced under different impregnation ratios of phosphoric acid to lignin, which showed that the BET increased with the increase in impregnation ratio. The lignin-based activated carbon produced with the impregnation ratio of 2.5:1 had the largest BET of 806.74 m^2^/g, which was used as the follow-up research object and named LAC.

**Figure 5:**
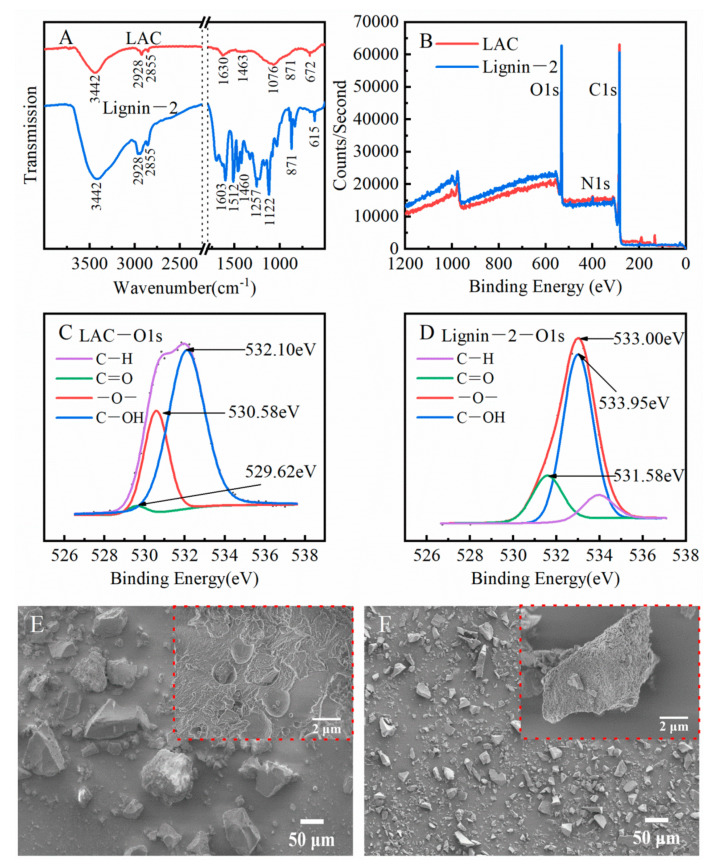
(**A**) FTIR spectra, (**B**–**D**) XPS spectra and (**E**,**F**) SEM of lignin-2 and LAC.

The structures of lignin-2 and LAC were revealed by FT-IR, XPS and SEM (see Figure 5). As shown in the FT-IR spectra (Figure 5A), the main signals appear at 1603, 1512 cm^−1^ (aromatic skeletal vibrations), and the absorption peak at 1460 cm^−1^ is attributed to the C-H deformation in –OCH_3_. The bands at 1122 and 871 cm^−1^ were prominent, indicating the presence of C-O and C=O [26]. Compared with lignin, the characteristic peak signals and intensities of LAC spectrum were significantly reduced, indicating that bonding bonds and functional groups on the lignin were largely destroyed during the high-temperature activation. However, some signals of oxygen-containing functional groups could still be observed, which contributed to the adsorption of heavy metal ions through chelation.

XPS results are shown in Figure 5B–D. Figure 5B shows that both lignin-2 and LAC had high C, O elements and few N elements, while LAC contained few P elements due to phosphoric acid activation. LAC had lower O element and higher C element than F4, which was attributed to the high-temperature activation. Moreover, for Figure 5C,D, the signals of lignin-2 XPS spectra at 533.95 eV, 533.00 eV, and 531.58 eV were caused by C-OH, -O-, C=O, and their contents are 10.24%, 68.78%, and 20.98%, respectively. The signals of LAC XPS spectra at 532.10 eV, 530.58 eV, 529.62 eV are attributed to C-OH, -O-, C=O, and their contents are 69.53%, 28.56%, and 1.91%, respectively [44]. By comparison, it could be concluded that the ether bond and C=O on lignin-2 were seriously destroyed in the process of high-temperature activation. Figure 5E,F show the surface morphological features of lignin-1 and LAC, respectively. The surface of lignin-2 was smooth and dense, with few deep pores. However, after carbonization and activation, large amounts of pores appeared on the surface of LAC, which was in line with BET results. A large number of pores would provide more adsorption sites for LAC to purify wastewater containing heavy metal ions.

### 2.6. Adsorption of LAC to Wastewater Containing Cu (II)

#### 2.6.1. Adsorption Isotherms and Adsorption Kinetics

The adsorption isotherm is often used to represent the relationship between the equilibrium concentration and the adsorption capacity, which is helpful for revealing the adsorption mechanism. The Langmuir model [45] and Freundlich model [46] were used to study the equilibrium of copper ions between liquid and solid phases; the detailed analysis process is shown in Text S1. In short, the adsorption process of copper ions by LAC was suitable for the Freundlich model, and the experimental maximum adsorption capacity (79.65 mg/L) was very close to the theoretically calculated maximum adsorption capacity. This result proved that the adsorption process belonged to multi-layer adsorption and heterogeneous adsorption, which was beneficial for improving the adsorption capacity of LAC.

The adsorption kinetics of LAC adsorption of copper ions was studied using two kinetic models derived from pseudo-first-order and pseudo-second-order models [47]; the detailed analysis process is shown in Text S2. The results showed that the R^2^ values of the first-order model and the second-order model were both greater than 99%, indicating that the adsorption of copper ions by LAC included both physical adsorption and chemical adsorption. This was a manifestation of the LAC with high adsorption capability.

#### 2.6.2. Effect of LAC Dosage

**Figure 6:**
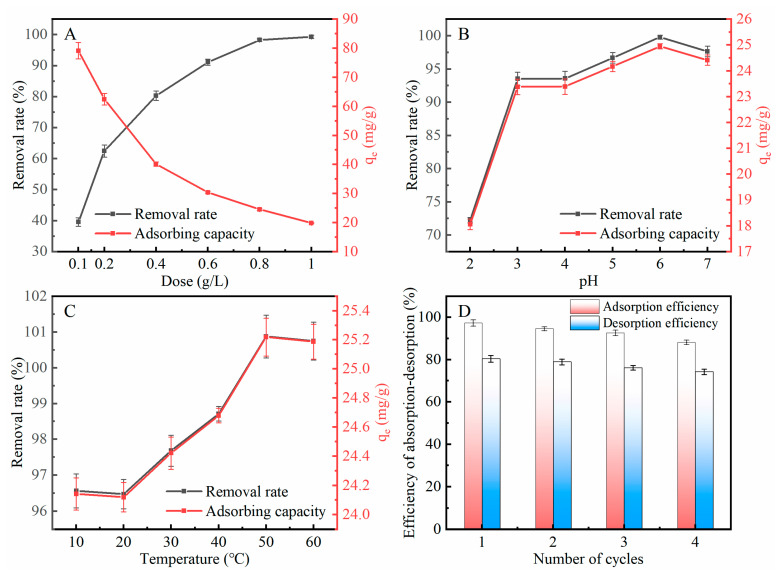
Effects of (**A**) dose, (**B**) wastewater pH, (**C**) temperature and (**D**) number of cycles on the removal rate and the adsorption capacity. Error bars represent the standard deviation from three repetitions.

From Figure 6A, the removal rate of Cu (II) increased gradually as the dosage increased, and the removal rate was over 98% at the LAC dosage of 0.8 g/L. Adsorption sites increased with larger dosage of LAC. Even if the concentration of Cu (II) in wastewater was quite low, enough adsorption sites could still effectively adsorb Cu (II). However, the adsorption capacity of LAC decreased gradually with increasing LAC dosage. When the dosage of LAC increased, Cu (II) was more easily adsorbed on the surface of LAC and many adsorption sites were not utilized, resulting in a lower adsorption capacity.

#### 2.6.3. Effect of Wastewater pH

The applicability of adsorbents to wastewater with different pH was an important factor to evaluate the quality of adsorbents [48]. The effect of pH on the adsorption performance of LAC is shown in Figure 6B. At increasing pH (from 2.0 to 3.0), the removal rate and adsorption capacity of LAC showed an upward trend. At the pH range 3.0–6.0, the removal rate and adsorption capacity of LAC gradually increased with the increase of wastewater pH. In a strong acid environment (pH = 2), the negatively charged functional groups on LAC were difficult to ionize, and a large number of hydrogen ions increased the electrostatic repulsion between LAC and copper ions, resulting in poor adsorption effect. With the increase of pH, the electrostatic repulsion between LAC and copper ions was weakened due to the reduction of hydrogen ions, and the adsorption effect was enhanced under the action of electrostatic attraction and chelation [49]. When the wastewater pH value was 6, the removal rate of copper ions by LAC was nearly 100%. However, as the wastewater pH value continued to increase, the adsorption effect of LAC decreased. This was because when the pH value was higher than 6, copper ions started to precipitate, which limited the adsorption effect of LAC. In general, LAC exhibited high adsorption capacity for copper ions over a wide pH range, except for strong acid environments.

#### 2.6.4. Effect of Temperature

The Brownian motion of copper ions and their diffusion on LAC surface and pore of were affected by temperature. Figure 6C shows that LAC had excellent adsorption effect on copper ions at all temperatures (10~60 °C). The adsorption effect increased at increasing temperature, proving that the process was endothermic. Moreover, higher temperature could increase the number of activated molecules on LAC and increase the contact probability between LAC and copper ions, improving the adsorption effect. However, the adsorption capacity of LAC decreased slightly when the temperature was higher than 50 °C. This was attributed to the excessively high temperature intensifying Brownian motion, which made the heavy metal ions on the adsorbent surface overactive. Some heavy metal ions were desorbed immediately before entering the adsorbent, thus inhibiting the progress of adsorption.

#### 2.6.5. Regeneration of LAC

The reproducibility and reusability of LAC are important parameters to measure its commercialization potential. In this study, the reproducibility and reusability of LAC were tested by four consecutive adsorption–desorption cycles, and the detailed procedures were described in Text S3. From Figure 6D, the cyclic investigation showed that adsorption efficiencies of LAC were 97.32%, 94.63%, 92.63%, and 88.12% from the four cycles, respectively, while the desorption efficiencies were 80.40%, 78.92%, 76.15%, and 74.26%, respectively. It could be found that both the adsorption and desorption efficiencies of LAC decreased after cycling, which was attributed to the hard-to-desorb copper ions and the aging of a small part of the adsorption sites [50]. Satisfyingly, after four cycles, LAC still had 88.12% adsorption efficiency and 74.26% desorption efficiency, indicating the outstanding reusability of LAC.

#### 2.6.6. Prospect of Industrialization

The technology proposed in this study has great prospects for large-scale production due to its advantages of easy operation, cost-effectiveness, and low pollution. Considering the industrialization cost of lignin fractionation, one-step ethanol fractionation of industrial lignin can achieve similar effects as above, reducing the difficulty and cost of fractionation. Moreover, the synthetic process of lignin-based flocculants in this paper avoided the use of a number of expensive, harmful solvents, such as dioxane, high-concentration alkaline solutions, etc., which is cost- and eco-efficient. It is worth noting that the recovery and reuse of ethanol from either fractionation processes or the preparation of lignin-based flocculants can be easily achieved in industrialization. Regarding the preparation process of lignin-based activated carbon, the carbonization process and the activation process are integrated, which simplifies the steps and reduces the cost. In particular, combining the technology in this study with the corn cob biorefinery process (production of xylose, ethanol) can greatly improve the utilization rate of biomass, characterized by environmental friendliness, cost-effectiveness, and ecological benefits.

## 3. Materials and Methods

### 3.1. Materials

The alkali lignin (KL) isolated from corn cob was supplied by Shandong Longlive Biotechnology Co., Ltd. (Dezhou, China). In short, corn cob was first subjected to hydrothermal treatment in order to degrade hemicellulose, and then the residue was treated with an alkaline solution (1.5% NaOH) for 3 h at 80 °C. Finally, the KL was obtained by adjusting the lignin-rich alkaline solution to acidity. N-butanol, ethanol, methanol, dioxane, trimethyl-2-methacroyloxyethylammonium chloride (METAC), azobisisobutyronitrile (AIBN), kaolin, and H_3_PO_4_ were purchased from Sinopharm Group Co., Ltd. ( Beijing, China).

### 3.2. Sequential Solvent Fractionation

**Scheme 2:**
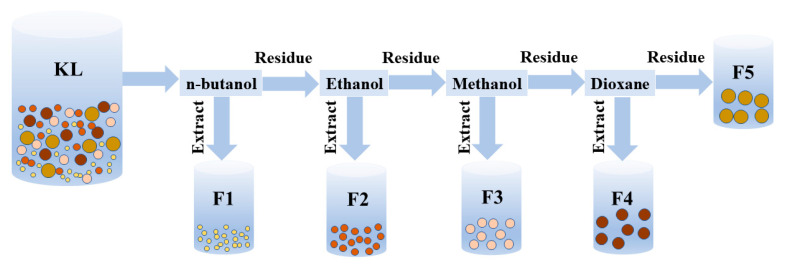
Scheme for organic solvent fractionation of KL.

As shown in Scheme 2, industrial alkali lignin (KL) from corn cob was fractionated sequentially using five recoverable organic solvents. The specific procedure in detail has been explained in our previous studies [23]. Finally, the lignin fractions collected from n-butanol, ethanol, methanol, and dioxane were referred as F1, F2, F3, and F4, respectively, and F5 was an insoluble residue.

### 3.3. Production of a Lignin-Based Flocculant

Based on full structural interpretation of all lignin, lignin-based flocculants were produced in ethanol solvent using lignin with high reactivity and accessibility as raw material, METAC as monomer, and AIBN as initiator. A certain proportion of lignin, METAC, and AIBN were added to the reactor. The reaction temperatures were 50, 60, 70 and 80 °C, respectively, for different times (2, 3, 4, and 5 h) under N_2_ condition. At complete reaction, the mixture was added dropwise to 3 volumes of acetone to collect the precipitate. The precipitate was then washed with acetone several times, and the pure lignin-based flocculant was gained after lyophilization. Referring to the aforementioned steps, PMETAC was produced by the conditions of 0.42 g AIBN, 14 g METAC, 4 h, and 60 °C.

### 3.4. Production of Lignin-Based Activated Carbon

The lignin consisting of high content of carbon was mixed with 85% phosphoric acid according to different impregnation ratios (phosphoric acid:F4 was 0.5:1~2.5:1), and nitrogen (500 mL/min) was used as protective gas for carbonization activation in a tubular furnace at 800 °C for 90 min. The obtained carbonized products were further purified by washing with hydrochloric acid, and followed by deionized water to adjust pH to 7.0. Finally, the carbonized products washed to neutral were dried at 105 °C to obtain lignin-based activated carbon.

### 3.5. Characterization

FT-IR of all samples was analyzed via a Brock tensor II spectrometer by the KBr pellet method; 2D-HSQC and ^31^P NMR were determined by a Bruker AVIII [51,52]. The determination methods of zeta potential and molecular weight of samples were described in previous studies [38,53]. The SEM micrographs were obtained by magnifying samples 200 and 10,000 times under 1.0 kV acceleration voltage using an emission scanning electron microscope (SU8020, Hitachi, Tokyo, Japan) [54]. XPS spectra of LAC and lignin-2 were recorded by the XASAM 800 spectrometer (Kratos Analytical, Manchester, UK) to analyze the content of elements in LAC and lignin-2 [44]. The specific surface analysis (BET) was determined by an ASAP 2020M (Micromeritics, Norcross, GA, USA) after degassing the dried samples at 200 °C for 7 h [55,56].

### 3.6. Flocculation Experiments

Flocculation experiments were performed using kaolin suspensions as simulated wastewater. Lignin-based flocculant was added into 100 mL kaolin suspension. After that, a mechanical stirring device was used to stir for 3 min (250 rpm), followed by 15 min (100 rpm), and finally the mixture stood for 90 min. The turbidity of kaolin suspension before and after flocculation was measured by WGZ-200S (Shanghai Xinrui Instrument Co., Ltd.), expressed by (T_0_) and (T_t_), respectively. The removal rate was calculated by the following formula:

Removal rate = (T_0_ − T_t_)/ T_0_ × 100% (1)

### 3.7. Adsorption Experiments

To a solution of Cu (II) solution (25 mL, 20 mg/L, pH = 5.8), LAC was added and then stirred for 120 min. After that, the supernatant was collected via a 0.45 μm membrane. Then the filtrate was measured by ICP (SPECTROBLUE ICP-OES, Spike Analytical Instruments, Germany) to determine the remaining Cu (II) content. The Cu (II) removal rate and the LAC adsorption capacity (q_e_) were calculated by the following formula:

removal rate = (C_0_ − C_t_)/C_0_ × 100% (2)

q_e_ = (C_0_ − C_t_) × V/D (3)

where C_0_ and C_t_ (mg/L) stand for the concentrations of Cu (II) before and after adsorption, respectively. V (mL) represented the solution volume of added Cu (II), and D (mg) indicated the dosage of LAC.

## 4. Conclusions

In order to produce lignin-based products more cleanly, industrial lignin was divided into several more homogeneous lignin fractions by organic solvent fractionation, and the structure of each fraction was adequately deciphered. Lignin-1 fractionated from ethyl acetate and ethanol had superior reactivity and reaction accessibility, and was used as raw material to produce lignin-based flocculant in green, inexpensive and recyclable ethanol solvents. Lignin-2 fractionated from methanol and dioxane had a high carbon content and was used as a precursor for the production of activated carbon. After optimizing the reaction conditions, LBF with removal rate of over 98% for kaolin suspension and LAC with 79.65 mg/g adsorption capacity for copper ion were successfully produced. The flocculation mechanism of LBF-treated kaolin suspension was charge attraction and bridging, and the adsorption of copper ions by LAC belonged to the multi-layer adsorption under the combined action of physical adsorption and chemical adsorption. Moreover, both LBF and LAC could be well applied to wastewater with complex external environment, showing great commercialization potential. The strategy of obtaining the desired lignin components by lignin fractionation and then making targeted high-value utilization of them is expected to be applied to the preparation of other lignin-based products. In addition, strengthening the structural interpretation of lignin and its derivatives, and establishing a stable structure–activity relationship are inevitable ways to develop lignin-based products in the future.

## Figures and Tables

**Table 1:** Yields and structure information of lignin samples.

Lignin Samples	Yield (%)	Mn (g/mol)	Mw (g/mol)	PDI	Total Phenolic−OH (mmol/g)	COOH (mmol/g)
KL		881	2777	3.15	4.53	2.72
F1	15.00	781	1141	1.46	4.97	3.12
F2	34.93	1885	2696	1.43	4.55	2.74
F3	19.10	2807	3987	1.42	3.17	1.56
F4	21.22	4355	6794	1.56	2.77	1.26
F5	9.15	4676	9820	2.10	2.30	1.21

## Data Availability

Not applicable.

## Supplementary Materials

The following supporting information can be downloaded at: https://www.mdpi.com/article/10.3390/ijms23126617/s1. References [57,58,59,60] are cited in the supplementary materials.
