# Supplementary material for: Customized Utilization Strategies of Industrial Lignin to Produce Adsorbents and Flocculants Based on Fractionation and Adequate Structural Interpretation
Source: Int J Mol Sci. 2022 Jun 14;23(12):6617. doi: 10.3390/ijms23126617 (PMC9223612; doi:10.3390/ijms23126617)
Supplement: Supplementary file 1 [file ijms-23-06617-s001.zip › ijms-1755378-supplementary.pdf]

International Journal of
Molecular Sciences

MDPI

Article: Supplementary Material

Customized Utilization Strategies of Industrial Lignin to Produce Adsorbents and Flocculants Based on Fractionation and Adequate Structural Interpretation

Lei Wang [1,*], Dewei Yang [1], Xiaohan Li [1], Xinyi Zhu [1], Jungang Jiang [1], Yifan Zhang [1], Xue Chen [2] and Hongbo Yu [3]

[1] Hubei Provincial Key Laboratory of Green Materials for Light Industry, Hubei University of Technology, Wuhan 430068, China; y13235634365@163.com (D.Y.); lxiaohan0317@163.com (X.L.); 102000414@hbut.edu.cn (X.Z.); jungang.jiang@hbut.edu.cn (J.J.); zhangyifan@hbut.edu.cn (Y.Z.)

[2] School of Engineering, Jining University, Qufu 273155, China; chenxue37121@163.com

[3] Key Laboratory of Molecular Biophysics of MOE, College of Life Science and Technology, Huazhong University of Science and Technology, Wuhan 430074, China; yuhongbo@hust.edu.cn

* Correspondence: wanglei@hbut.edu.cn

Text S1. Adsorption isotherms

The adsorption isotherms are often used to describe the relationship between the equilibrium adsorption capacity (q_e, mg/g) and the equilibrium concentration (C_e, mg/L), which is helpful for revealing the adsorption mechanism [1]. In this study, two isotherm models, such as Langmuir (Equation 1) and Freundlich (Equation 2) models [2,3], were used to study the equilibrium of copper ions between liquid and solid phases:

$$C_e/q_e = C_e/q_m + 1/(K_L \times q_m) \tag{1}$$

$$\lg q_e = \lg K_F + (1/n) \times \lg C_e \tag{2}$$

Where q_e (mg/g) is the sorption capacity at equilibrium, and C_e (mg/L) is Cu (II) concentration at equilibrium. q_m is the saturation capacity of the sorbent (mg/g), K_L is the adsorption constant (L/mg), K_F [(mg/g) (L/mg)$^{1/n}$] is a Freundlich constant related to the relative adsorption capacity and n is an empirical parameter indicating adsorption intensity. As shown in Figure S3C, the solid points represent the experimental data and the solid lines are the fit lines. The parameters of the adsorption isotherm model were listed in the Table S3. The R^2 value (0.9808) of the Freundlich model was greater than the R^2 value (0.9208) of the Langmuir model, proving that the adsorption process of LAC was more suitable to the Freundlich model, and LAC exhibited a maximum adsorption capacity up to 79.65 mg/g. This result proved that the adsorption of copper ions by LAC belonged to multi-layer adsorption and heterogeneous adsorption, which was beneficial to the improvement of the adsorption capacity of LAC [4].

Text S2. Adsorption kinetics

The adsorption kinetics of LAC adsorption of copper ions was studied using two kinetic models, such as pseudo-first-order (Equation 3) and pseudo-second-order (Equation 4) [5]:

$$\lg(q_e - q_t) = \lg q_e - k_1 \times t \tag{3}$$

$$t/q_t = 1/(k_2 \times q_e^2) + t/q_e \tag{4}$$

Where q_t (mg/g) is the amount adsorbed at time t (min), and q_e (mg/g) denotes the amount adsorbed at equilibrium, k_1 (min^{-1}) and k_2 (g/(mg min)) represent the adsorption rate constants. The results were shown in Figures S3B and D. The calculated values of kinetic parameters and correlation coefficients were presented in Table S3. The R^2 values of the first-order model and the second-order model were both greater than 99%, indicating that the adsorption of copper ions by LAC included both physical adsorption and chemical adsorption [6]. This was also one of the reasons for the efficient adsorption capacity of LAC.

Text S3. LAC cycle adsorption

25 mL Cu (II) aqueous solution (20 mg/L, pH = 5.8) and 20 mg LAC were added to the flask. Stirred the mixture at room temperature for 120 min. After 120 min, the liquid was filtered through 0.45 μm membrane to collect the liquid. Then the filtrate was analyzed by ICP to determine the content of remaining Cu (II). In order to study the reusability of LAC, the absorption and adsorption cycle experiments were carried out. For desorption experiments, LAC loaded with Cu (II) in Step 1 was transferred to 50 mL 0.1 mol/L HCl solution and stirred at 150 rpm at room temperature for 2 h. Cu (II) concentration in solution could also be determined by ICP to judge the degree of desorption. After desorption, the LAC was repeatedly cleaned to neutral with deionized water and then dried to constant weight at 105 °C to repeat Step 1 (this cycle was repeated 4 times) [7].

Table S1. The preparation conditions for LBFs.

Sample number	METAC/Lingin-1 mass ratio	ABIN dose (g)	Temperature (°C)	Time (h)	Mw (g/mol)	Mn (g/mol)	PDI
1	2	0.18	60	4	326480	238300	1.37
2	4	0.3	50	4	384930	253240	1.52
3	4	0.3	60	4	471590	323010	1.46
4	4	0.3	70	4	426480	346730	1.23
5	4	0.3	80	4	376810	234040	1.61
6	4	0.3	60	2	293470	206680	1.42
7	4	0.3	60	3	376420	283020	1.33
8	4	0.3	60	5	478660	325610	1.47
9	6	0.42	60	4	483640	350460	1.38
10	8	0.54	60	4	452460	353480	1.28

Table S2. Surface area of lignin-2 and LACs.

Samples	Immaculate ratio (phosphoric acid: lignin)	S_{BET} (m^2/g)
lignin-2	/	5.45
LAC-1	0.5:1	503.68
LAC-2	1.5:1	637.97
LAC	2.5:1	806.74

Table S3. Parameters of adsorption isotherm models, pseudo-first-order and pseudo-second-order kinetic model for the adsorption of Cu (II) on LAC.

Langmuir			Freundlich		
q_m (mg/g)	K_L (L/mg)	R^2	K_F[(mg/g)(L/mg)$^{1/n}$]	n	R^2
80.46	0.07	0.9208	23.2	4.47	0.9808
Pseudo-first-order			Pseudo-second-order		
k_1(min^{-1})	q_e(mg/g)	R^2	k_2(g/(mg min))	q_e(mg/g)	R^2
0.19	24.37	0.9987	0.05	24.70	0.9993

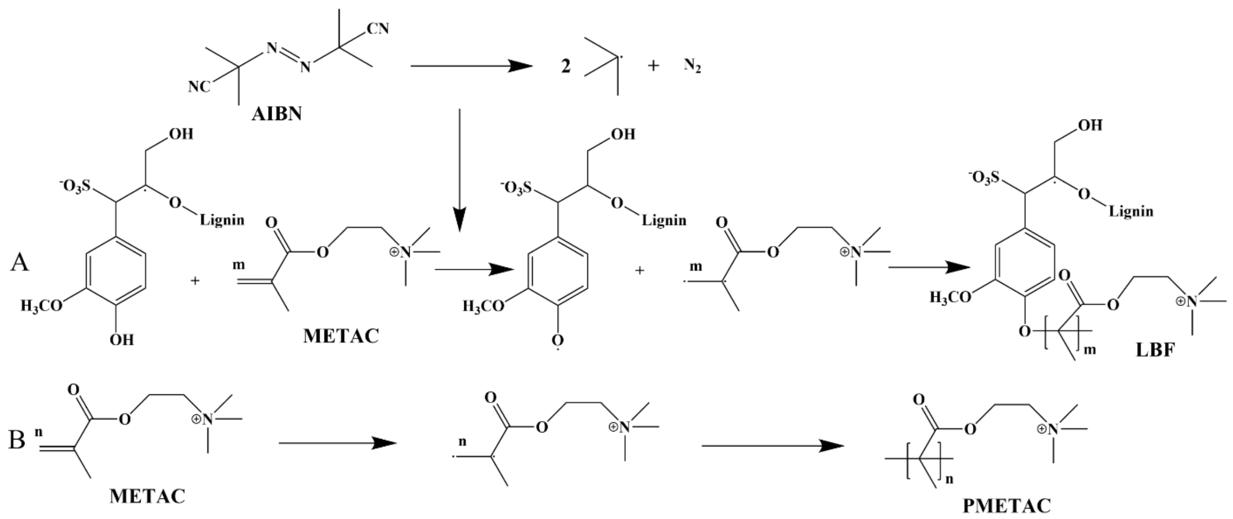

Scheme S1. Polymerization schemes of the copolymerization of lignin with METAC, and homopoly-mer PMETAC.

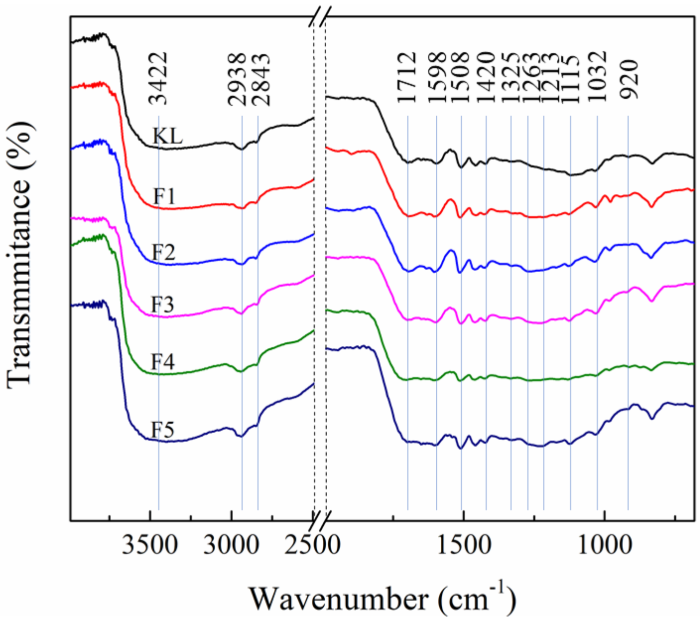

Figure S1. FTIR spectra of KF and fractionated lignins.

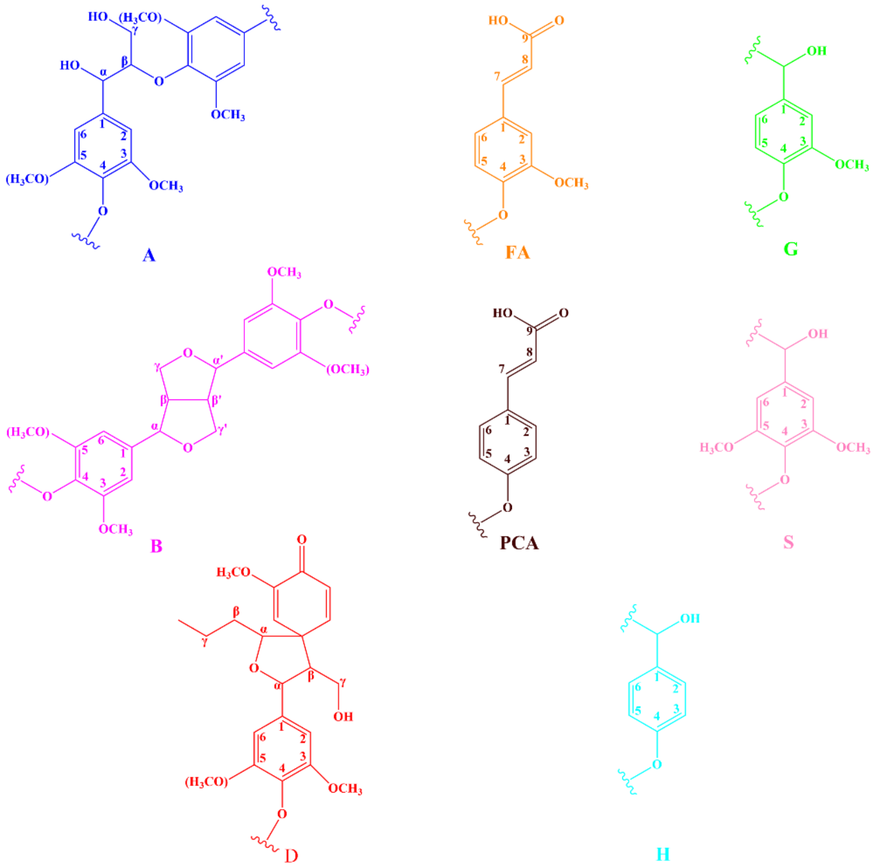

Figure S2. (**A**) β-*O*-4 alkyl-aryl ethers; (**B**) resinols; (**D**) spirodienone substructures; (**FA**) ferulic acid; (***P*CA**) *p*-coumaric acid; (**H**) *p*-hydroxyphenyl units; (**G**) guaiacyl units; (**S**) syringyl units.

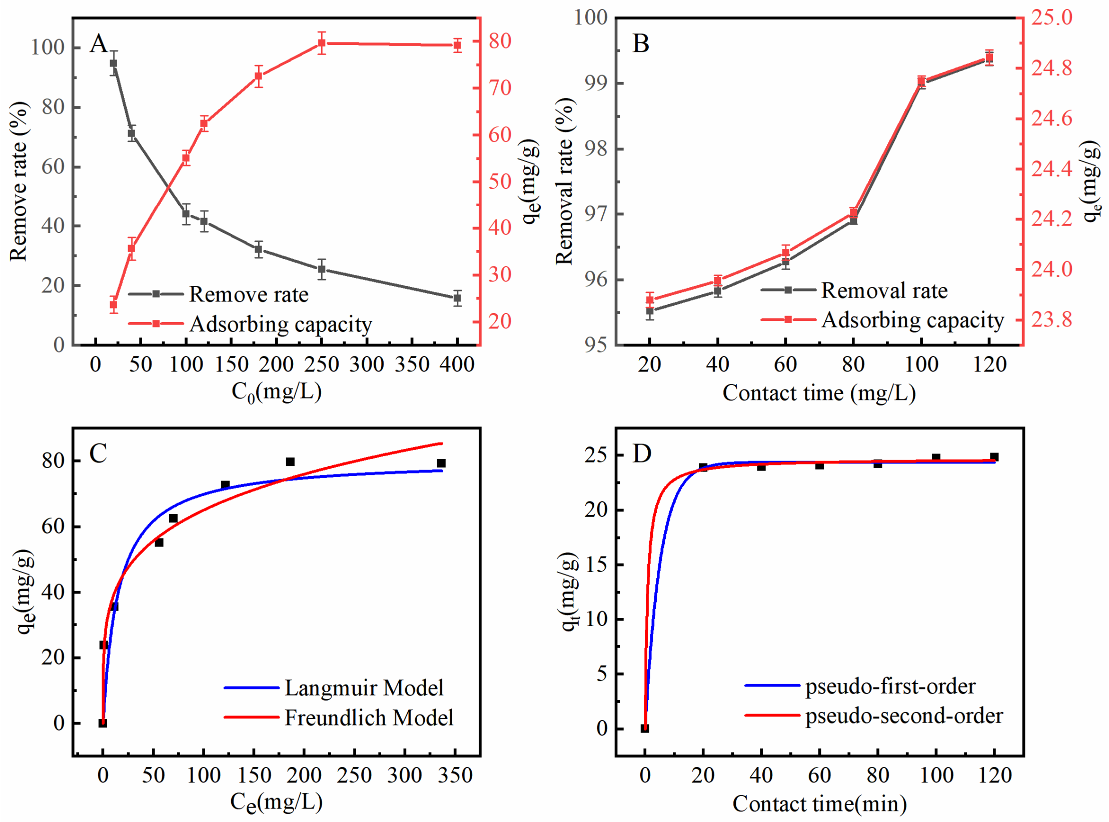

Figure S3. Effects of (**A**) initial concentration of copper ions, (**B**) contact time on the removal rate and the adsorbance. (**C**) Adsorption isotherms of Cu (II) on LAC, and fitting by Langmuir and Freundlich. (**D**) Adsorption kinetics of Cu (II) by LAC and fitting by pseudo-second-order model and pseudo-first-order model.

References

1. Wang, Y.; Wang, H.; Peng, H.; Wang, Z.; Wu, J.; Liu, Z. Dye adsorption from aqueous solution by cellulose/chitosan composite: Equilibrium, kinetics, and thermodynamics. *Fibers Polym.* **2018**, *19*, 340–349.
2. Langmuir, I. The adsorption of gases on plane surfaces of glass, mica and platinum. *J. Am. Chem. Soc.* **1918**, *40*, 1361–1403.
3. Freundlich, H. Over the adsorption in solution. *J. Phys. Chem* **1906**, *57*, 1100–1107.
4. Lakey, P.S.; Eichler, C.M.; Wang, C.; Little, J.C.; Shiraiwa, M. Kinetic multi-layer model of film formation, growth, and chemistry (KM-FILM): Boundary layer processes, multi-layer adsorption, bulk diffusion, and heterogeneous reactions. *Indoor Air* **2021**, *31*, 2070–2083.
5. Lagergren, S. Zur theorie der sogenannten adsorption geloster stoffe. *K. Sven. Vetensk. Handl.* **1898**, *24*, 1–39.
6. Al-Ghouti, M.A.; Da'ana, D.A. Guidelines for the use and interpretation of adsorption isotherm models: A review. *J. Hazard. Mater.* **2020**, *393*, 122383.
7. Wang, X.; Li, X.; Peng, L.; Han, S.; Hao, C.; Jiang, C.; Wang, H.; Fan, X. Effective removal of heavy metals from water using porous lignin-based adsorbents. *Chemosphere* **2021**, *279*, 130504.
